# New Insights into Alzheimer's Disease Progression: A Combined TMS and Structural MRI Study

**DOI:** 10.1371/journal.pone.0026113

**Published:** 2011-10-12

**Authors:** Eini Niskanen, Mervi Könönen, Sara Määttä, Merja Hallikainen, Miia Kivipelto, Silvia Casarotto, Marcello Massimini, Ritva Vanninen, Esa Mervaala, Jari Karhu, Hilkka Soininen

**Affiliations:** 1 Department of Applied Physics, University of Eastern Finland, Kuopio, Finland; 2 Department of Clinical Radiology, Kuopio University Hospital, Kuopio, Finland; 3 Department of Clinical Neurophysiology, Kuopio University Hospital, Kuopio, Finland; 4 Institute of Clinical Medicine, Neurology, University of Eastern Finland, Kuopio, Finland; 5 Aging Research Center, Karolinska Institutet, Stockholm, Sweden; 6 Department of Clinical Sciences “L. Sacco”, Università degli Studi di Milano, Milan, Italy; 7 Institute of Clinical Medicine, Clinical Radiology, University of Eastern Finland, Kuopio, Finland; 8 Institute of Clinical Medicine, Clinical Neurophysiology, University of Eastern Finland, Kuopio, Finland; 9 Nexstim Ltd, Helsinki, Finland; 10 Department of Physiology, University of Eastern Finland, Kuopio, Finland; 11 Department of Neurology, Kuopio University Hospital, Kuopio, Finland; University of California, San Francisco, United States of America

## Abstract

**Background:**

Combination of structural and functional data of the human brain can provide detailed information of neurodegenerative diseases and the influence of the disease on various local cortical areas.

**Methodology and Principal Findings:**

To examine the relationship between structure and function of the brain the cortical thickness based on structural magnetic resonance images and motor cortex excitability assessed with transcranial magnetic stimulation were correlated in Alzheimer's disease (AD) and mild cognitive impairment (MCI) patients as well as in age-matched healthy controls. Motor cortex excitability correlated negatively with cortical thickness on the sensorimotor cortex, the precuneus and the cuneus but the strength of the correlation varied between the study groups. On the sensorimotor cortex the correlation was significant only in MCI subjects. On the precuneus and cuneus the correlation was significant both in AD and MCI subjects. In healthy controls the motor cortex excitability did not correlate with the cortical thickness.

**Conclusions:**

In healthy subjects the motor cortex excitability is not dependent on the cortical thickness, whereas in neurodegenerative diseases the cortical thinning is related to weaker cortical excitability, especially on the precuneus and cuneus. However, in AD subjects there seems to be a protective mechanism of hyperexcitability on the sensorimotor cortex counteracting the prominent loss of cortical volume since the motor cortex excitability did not correlate with the cortical thickness. Such protective mechanism was not found on the precuneus or cuneus nor in the MCI subjects. Therefore, our results indicate that the progression of the disease proceeds with different dynamics in the structure and function of neuronal circuits from normal conditions via MCI to AD.

## Introduction

Alzheimer's disease (AD) is the most common disease causing dementia. It has been predicted that in 2050 the number of AD patients worldwide will have quadrupled from the level of 2006 to 106 million AD patients, meaning that 1 out every 85 persons will suffer from AD [1]. A thorough understanding of the underpinnings of the disease process is essential in searching new treatments for AD. Therefore, a combination of structural and functional information of the human brain could provide more detailed information of the influence of the neurodegenerative diseases on cortical brain areas.

Transcranial magnetic stimulation (TMS) of the motor cortex can evaluate different aspects of cortical excitability. In particular, the resting motor threshold (rMT) has been shown to reliably reflect cortical excitability [2]–[4]. Furthermore, rMT is altered in certain diseases such as vascular dementia [5], Parkinson's disease [6], juvenile myoclonus epilepsy [7] and progressive myoclonus epilepsy EPM1 [8]. In AD, several studies have described increased motor cortex excitability as compared to controls [9]–[13]. This hyperexcitability has been hypothesized to reflect impairment in cholinergic activity and a deficit of N-methyl-D-aspartic glutamate receptors [13]–[15].

The rMT, however, is highly dependent on the coil-cortex distance when expressed as the percentage of the maximal cortical stimulator output [16]–[18]. Therefore, instead of using the rMT to assess the motor cortex excitability, the corresponding value of the electrical field, EF_MT_, induced on the cortex by the magnetic stimulation should be used. Unfortunately, the true induced electric field is not measurable noninvasively. However, the induced electric field can be estimated based on the stimulation intensity, coil orientation and characteristics, and shape of the subject's head. Furthermore, the individual coil-cortex distance can be taken into account in the estimation of EF_MT_ by utilizing the subject's individual MRIs [19], [20]. Thus, by using EF_MT_, the assessment of cortical excitability can be based on purely neuronal basis irrespective of the distance between the stimulation coil and cortex.

Previously, cortical thickness analysis on AD patients has revealed cortical thinning in several brain areas known to be affected by AD neuropathology [21]–[23] and this thinning has been shown to be related to the clinical severity of AD, even in the early stage of the disease [24]. Furthermore, cortical thickness analysis has been proposed to have diagnostic utility in differentiating various neurodegenerative diseases and their variants, and perhaps also in predicting the progression from MCI to AD i.e. due to the different thickness profiles [25], [26]. Other widely used techniques to study brain atrophy in vivo are the different MRI volumetric methods, with voxel-based morphometry (VBM) being one of the most widely used techniques [27]. VBM, however, does not provide information about brain atrophy at the single-subject level i.e. it permits only group-level analysis. Cortical thickness analysis provides grey matter thickness information both in the subject's native space and in standard stereotactic space. This feature makes cortical thickness analysis suitable for both general group-level analysis and also for individual diagnostic purposes.

There are very few studies combining functional information of cortical excitability with structural information of brain. Using diffusion tensor imaging (DTI) rMT has been correlated with the fractional anisotropy of the white matter underlying the primary motor and premotor areas [18], [28]. Furthermore, by combining VBM results with rMT, it was found that the age-related volumetric findings such as increased cerebrospinal fluid volume could be associated with lower rMT [29]. Relative changes in grey matter density have been correlated with the changes in cortical excitability in subjects suffering from writer's cramp [30]. However, no direct comparison of the cortical grey matter thickness and the cortical excitability has ever been performed.

Previously, it has been suggested that neuronal loss might be one of the reasons responsible for motor cortex hyperexcitability in AD patients [15], [31]. The aim of this study was to examine this hypothesis by combining the cortical thickness analysis revealing the neuronal loss with the cortical excitability based on the EF_MT_. This relationship between structure and function of the brain was assessed in patients with AD, in individuals with mild cognitive impairment (MCI) and in age-matched healthy controls.

## Materials and Methods

### Participants

Originally 17 patients with AD and 21 patients with MCI were recruited into this study from population-based databases along with 22 healthy controls as indicated in detail in [32], [33]. Two of the AD patients, three of the MCI patients and a control were later excluded because of poor MRI quality. Therefore, the final sample was 15 AD patients, 18 MCI patients and 21 controls. There was no significant difference in age between the groups. More detailed patient demographics are presented in Table 1. The AD patients were diagnosed using the NINCDS-ADRDA criteria [34] which included an evaluation of medical history, physical and neurological examinations performed by a physician, and a detailed neuropsychological evaluation. Furthermore, other possible pathologies to account for the symptoms were excluded by MRI assessment, cerebrospinal fluid (CSF) analysis, electrocardiography, chest radiography, screening for hypertension and depression, and blood tests. Majority of the AD patients (12/15) were under acetylcholinesterase inhibitor (AChEI) medication. The MCI patients were diagnosed using the original criteria of the Mayo Clinic Alzheimer's Disease Research Center: 1) memory complaint by patient, family, or physician; 2) normal activities of daily living; 3) normal global cognitive function; 4) objective impairment in memory or in one other area of cognitive function as evident by scores > 1.5 S.D. below the age appropriate mean; 5) CDR score of 0.5; and 6) absence of dementia [35], [36]. Controls showed no impairment in detailed neuropsychological evaluation. All of the subjects' MR images were further read by an experienced neuroradiologist to exclude subjects with significant white matter hyperintensities or other structural abnormalities.

**Table 1:** Detailed subject demographics as well as electric field values (EF_MT_) corresponding to the resting motor threshold of thenar musculature, and ROI cortical thicknesses for each group.

Group	N (males/females)	Age (years)	MMSE ††	Handedness (right/left/ambidextrous)	EF_MT_ (V/m)	Cortical thickness (mm)		
						M1/S1†	Precuneus	Cuneus
AD	15 (5/10)	73.7±7.5	18.9±4.1	14/1/-	89.7±17.4	2.3±0.3	3.1±0.4	2.7±0.2
MCI	18 (9/9)	71.6±8.0	23.7±2.7	18/-/-	91.1±21.3	2.4±0.4	3.3±0.3	2.8±0.3
Controls	21 (10/11)	71.9±5.9	28.4±1.4	19/1/1	87.8±11.9	2.5±0.4 *	3.3±0.2	2.8±0.2

For each parameter, except N and handedness, the group mean and standard deviation are presented.

†† Significant difference among groups, (Kruskal-Wallis), *p<0.001* and between groups in all pair-wise comparisons (Mann-Whitney), *p<0.001*

† Significant difference among groups, (Kruskal-Wallis), *p<0.05*

* Significantly different from AD, (Mann-Whitney), *p<0.05*

### Ethics Statement

Written informed consent was obtained from all participants according to the Declaration of Helsinki. The study was approved by the Research Ethics Committee, Hospital District of Northern Savo.

### Magnetic resonance imaging

A T1-weighted structural MR image was acquired for all subjects in order to determine the cortical thickness and to provide the anatomical information in the navigated TMS study. The MR image was acquired with a 1.5 T MR scanner (Magnetom Avanto, Siemens AG, Erlangen, Germany) with the 3D-MPRAGE (magnetization-prepared rapid acquisition gradient echo) sequence using the following parameters for most of the subjects (N = 51): TR = 2400ms, TE = 3.5 ms, TI = 1000 ms, flip angle = 8°, 160 sagittal slices, voxel size =  1.2 mm × 1.2 mm × 1.2 mm. However, for cost saving reasons, the subjects' earlier 3D-MPRAGE images that were scanned within a year were used if they were available and had been scanned with satisfactorily good spatial resolution. Earlier scans with different imaging parameter sets were used for two controls (first set: TR = 1980 ms, TE = 3.93 ms, TI = 1100 ms, flip angle = 15°, 176 sagittal slices, voxel size =  1 mm × 1 mm × 1 mm, second set: TR = 9700 ms, TE = 4000 ms, TI = 300 ms, flip angle = 12°, 128 coronal slices, voxel size =  1 mm × 2 mm × 1 mm), and for one MCI subject (TR = 1900 ms, TE = 2.94 ms, TI = 1100 ms, flip angle = 15°, 160 coronal slices, voxel size =  0.5 mm × 1.5 mm × 0.5 mm). The average time from the MRI scanning to the TMS procedure was 2.4±4.0 months.

### Transcranial magnetic stimulation

TMS setup consisted of eXimia navigation system combined with a magnetic stimulator and a biphasic figure-of-eight TMS coil with a mean wing radius of 50 mm (eXimia NBS System and TMS Stimulator, Nexstim, Helsinki, Finland). The eXimia navigation system utilizes the subject's own MR image combining the anatomical information with online infrared-tracking of the subject's head and the TMS coil. This enables positioning the stimulation coil correctly on the scalp to stimulate the targeted cortical structure with an accuracy of 6 mm (for details, see [37]). The eXimia navigation software estimates the magnetic stimulation induced electric field on the cortex by analysing the information of the spherical head model that accounts for the local variation in the head curvature and the coil geometry as well as the location and orientation of the coil with respect to the head, and the electrical characteristics of the stimulation pulse [19]. The estimated electrical field is visualized on the reconstructed cortical surface at a selected depth from the scalp. Thus, the location of the maximum electric field on the surface is based on mathematical modelling and not only a projection of a virtual rod from the TMS coil.

**Figure 1:**
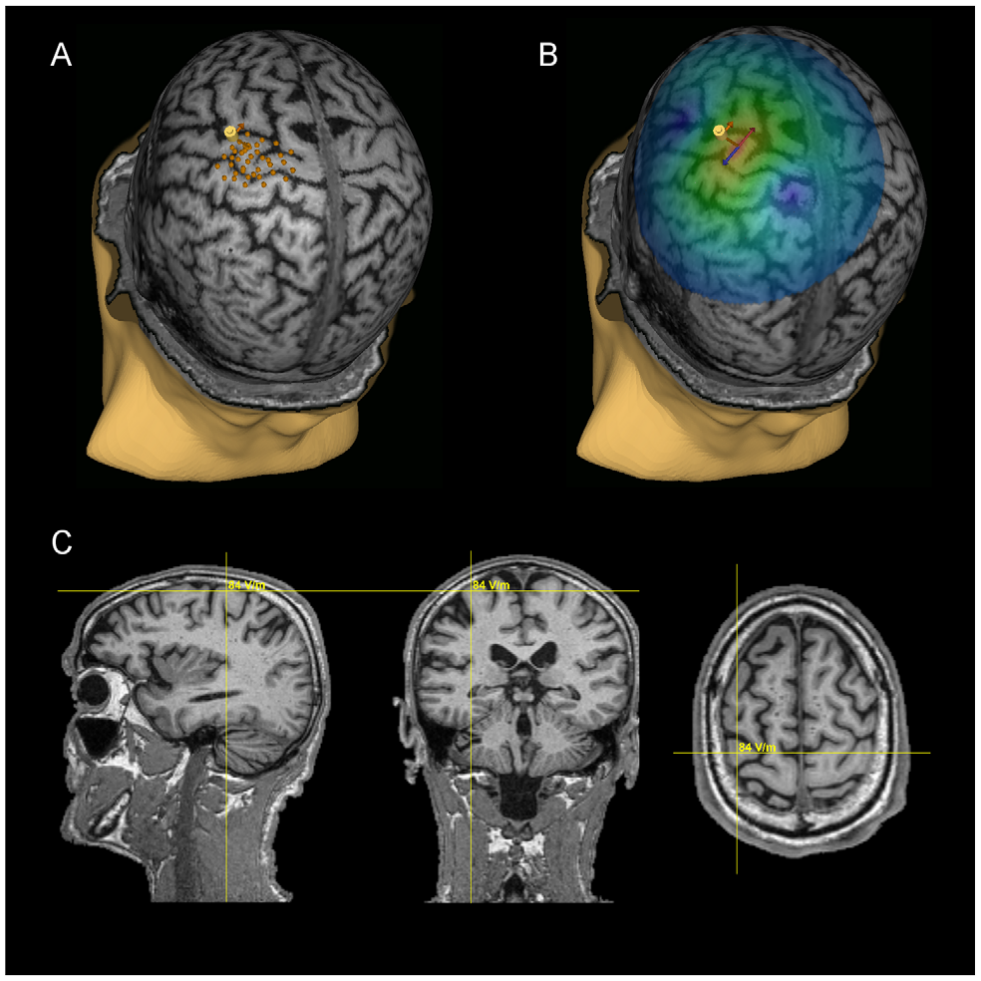
TMS measurements in a representative subject. A) The area around the anatomically defined hand-knob was extensively mapped to find the optimal stimulation area of the thenar muscle. The orange dots illustrate the stimulation locations for mapping and the yellow brick shows the coil orientation for the optimal stimulation site. B) The software estimates the induced electrical field on the surface and visualizes it with colors. C) The electrical field value corresponding to the resting motor threshold is shown numerically on top of the sagittal, coronal and axial slices. The crosshair shows the optimal stimulation location for the thenar muscle on the surface between grey and white matter.

The mapping procedure was done similarly as explained in detail in [38]. During magnetic stimulation, surface electromyography (EMG) was recorded and monitored continuously on-line (ME 6000, Mega Electronics Ltd., Kuopio, Finland). The active electrode was attached to the skin overlying the thenar muscle (abductor pollicis brevis) and the reference electrode on the first metacarpophalangeal joint. In order to locate the optimal stimulation site for the thenar muscle, the hand muscle area around the anatomically defined cortical “hand knob” [39] was extensively stimulated (see Figure 1A). The mapping was started near the hand knob and was continued in both lateral-medial and anteroposterior direction as far as measurable MEPs were elicited. To ensure the relaxation of the muscle, the background EMG activity was visually monitored during mapping. During stimulation, the coil was kept tangential to the surface of the head and the direction of the induced current perpendicular to the anatomically defined central sulcus. The location where TMS evoked the largest MEP was defined as the optimal stimulation site. The resting motor threshold i.e. the minimum TMS intensity that was sufficient to elicit MEPs with amplitude of at least 50 µV in five out of ten trials [2] was determined at the optimal stimulation site (see Figure 1B) and the corresponding electric field value EF_MT_ at the surface between the white matter (WM) and the gray matter (GM) was taken into the statistical analysis (see Figure 1C).

### Cortical thickness analysis

The cortical thickness was determined using the pipelining method developed at the McConnell Brain Imaging Centre, Montreal Neurological Institute, McGill University, Montreal, Canada [40]. The MR images were first registered to the standard ICBM152 template [41], intensity non-uniformities were corrected using the N3-algorithm [42], and the final brain mask was created [43]. The brain was segmented into GM, WM and cerebrospinal fluid (CSF) with the INSECT-algorithm [44] and the surfaces between the WM and GM as well as between GM and CSF were estimated using the CLASP-algorithm [45], [46]. The cortical thickness was determined between these two surfaces utilizing the t-link metric. Finally, the cortical thickness maps were smoothed with a 20mm FWHM kernel to increase the signal-to-noise ratio [45], [46].

### Statistical analysis

Whole brain correlation analysis between the EF_MT_ and the cortical thickness was performed on the left hemisphere in all subjects using an in-house written Matlab script (R2007b, The Mathworks Inc.) with an ANCOVA model with four covariates of no interest: age, gender, the volume of the MRI voxel to take into account the different MRI parameter settings, and the time difference between the MRI scan and the TMS procedure. Both negative and positive correlations were checked. The areas where there were significant negative correlations (*p<0.05*, corrected for multiple comparisons with false discovery rate (FDR) method, cluster minimum of 100 nodes) were further entered into a region-of-interest (ROI) analysis in which the mean cortical thickness in each ROI was calculated for each subject. Pearson correlation of the EF_MT_ and the mean ROI thickness within each group were calculated with SPSS (IBM SPSS Statistics, version 17.0, IBM Corporation, Somers, NY) to provide more detailed information of each ROI in the different study groups.

## Results

The group averages of EF_MT_ corresponding to each subject's rMT of the thenar muscle are presented in Table 1. There was no statistically significant difference between the groups in the EF_MT_, although the MCI subjects had a tendency towards higher EF_MT_ than the AD subjects and controls.

**Figure 2:**
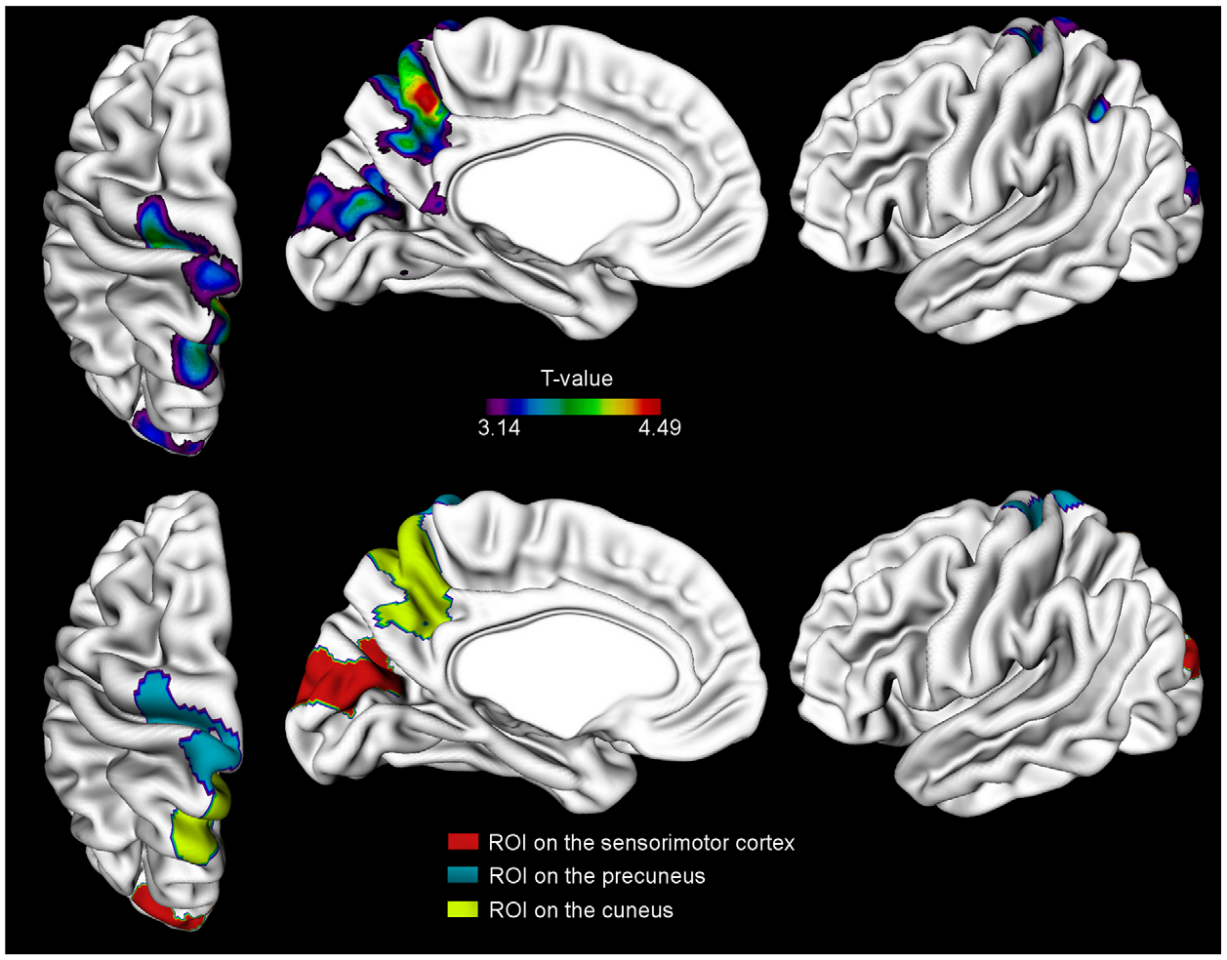
The areas of significant negative correlation between cortical thickness and EF_MT_ for all subjects (*p<0.05*, FDR-corrected) (upper row) and the corresponding regions of interest with a cluster minimum of 100 nodes (lower row). ROI 1 includes areas on M1 and S1 (in blue), ROI 2 encompasses the precuneus (in yellow) and ROI 3 in the cuneus (in red).

EF_MT_ was further correlated point-by-point with the cortical thickness on the left hemisphere in order to locate areas where there was a regional correspondence between cortical excitability and the amount of grey matter. This correlation analysis was performed on all subjects as one group. EF_MT_ correlated negatively with cortical thickness in areas on the sensorimotor cortex, on the precuneus and on the cuneus. A negative correlation means that the thinner the cortex, the stronger the stimulation intensity needed to produce MEPs. The correlation map is presented in Figure 2 (upper row). No significant positive correlations were found.

Based on the whole-hemisphere correlation analysis of all of the subjects' pooled data, three ROIs showing significant correlation with motor cortex excitability were found: a ROI containing the significant cluster on the sensorimotor cortex, a second ROI on the precuneus and a third ROI on the cuneus. The ROIs are illustrated in Figure 2 (lower row). The group mean thicknesses of each ROI are presented in Table 1. In all ROIs, the cortex was thinnest in the AD group. The mean thickness of the ROI on the sensorimotor cortex differed significantly among the groups (Kruskal-Wallis test, *p<0.05*).

**Figure 3:**
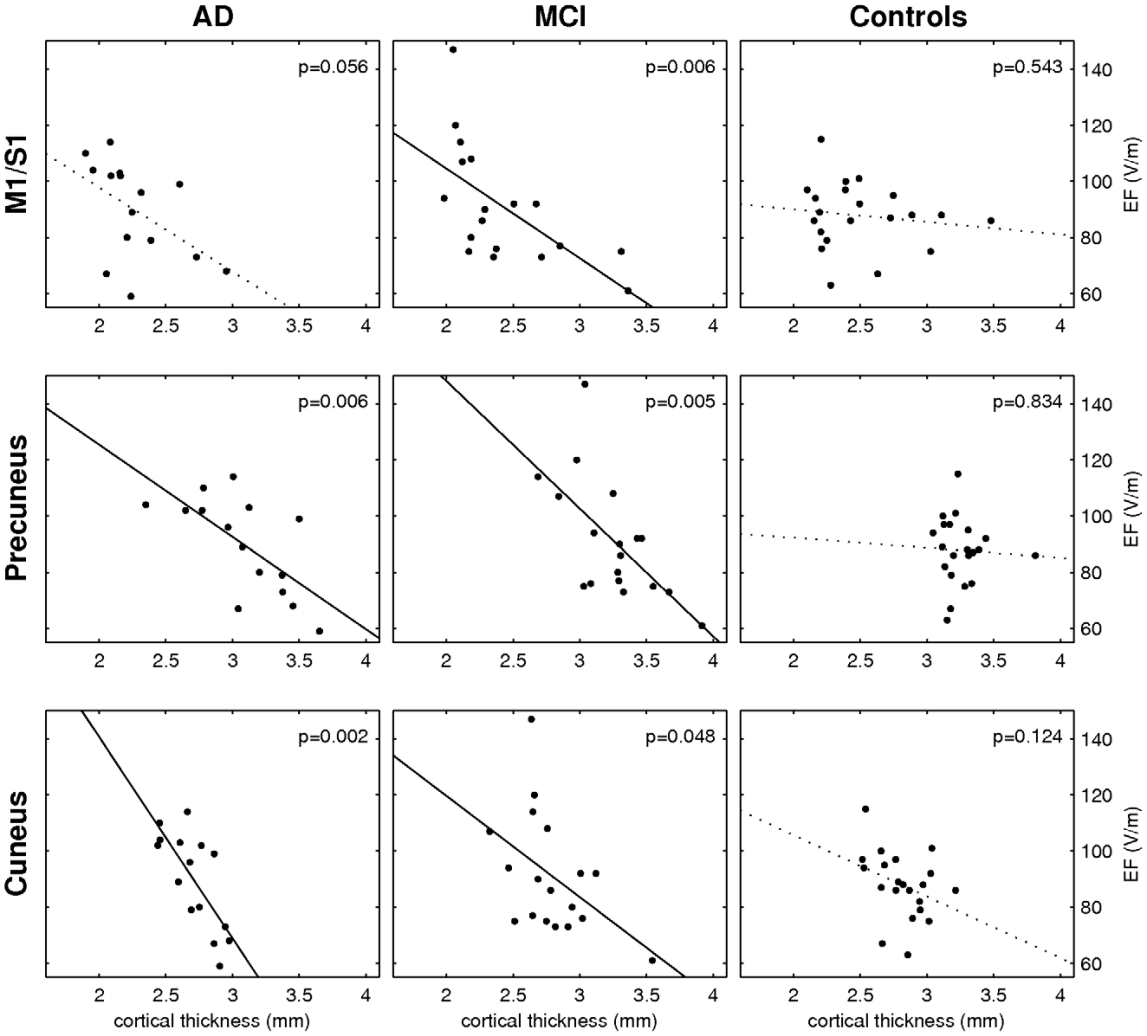
Scatter plots of the relationships between EF_MT_ and mean cortical thickness for AD patients (left column), MCI patients (middle column) and controls (right column) for M1/S1 on the first row, for the precuneus on the second row, and for the cuneus on the third row. The corresponding significance of the correlation is presented in the top right corner of each plot. Statistically significant (*p<0.05*) correlations are presented with solid regression lines.

ROI correlation analyses were further performed for each subject group separately. On the sensorimotor cortex, only MCI patients exhibited a significant negative correlation between EF_MT_ and cortical thickness (*p = 0.006*) although the correlation in AD subjects was only barely above the threshold (*p = 0.056*). On the precuneus, both AD subjects (*p = 0.006*) and MCIs showed a significant correlation (*p = 0.005*). On the cuneus, the AD group and the MCIs exhibited a significant correlation (*p = 0.002* and *p = 0.048*, respectively). The correlation between the EF_MT_ and the cortical thickness was not significant in controls in any of the ROIs. The scatter plots of the cortical thickness and EF_MT_ for each group are presented in Figure 3.

## Discussion

In this work, the relationship between the cortical thickness and motor cortex excitability was studied in patients with AD or MCI and in healthy controls. A significant negative correlation was found between the cortical thickness and EF_MT_ on the sensorimotor cortex, on the precuneus and on the cuneus. The relationship appeared to differ between the study groups depending on the cortical area: in AD subjects the correlation was weakest on the sensorimotor cortex whereas in MCI subjects on the cuneus. In healthy controls the EF_MT_ did not correlate with the cortical thickness in any of the ROIs.

Resting motor threshold along with the EF_MT_ reflects the excitability of a central core of neurons and combines the excitability of individual neurons with their local density (for a review, see [47]). In more details, the rMT depends on the excitability of cortico-cortical axons and is influenced by the diameter, coherence and density of these axons that connect the stimulated motor cortex with other cortical areas [28]. TMS given at intensity close to the rMT predominantly evokes I waves which are considered to arise from transsynaptic activation of corticospinal neurons [48]. By increasing the intensity of the magnetic stimulus, both the maximum of the induced electrical field and the size of the directly influenced brain volume increase. The amplitude of a MEP is known to increase with increasing stimulus intensity, evidence that stronger stimuli recruit more corticospinal neurons [49]. Therefore, in order to produce a measurable MEP, a sufficient number of neurons must be excited. This can be achieved by increasing the stimulation intensity because the increase of intensity activates neurons in a larger area and at the same time activates also those neurons with a higher activation threshold. Moreover, the stimulating current is most effective and stable when directed according to local columnar structure, i.e., perpendicular to the central sulcus, increasing the net number of synchronously activated neurons. Our results are in line with this proposal since the cortical thickness on the sensorimotor cortex was found to correlate negatively with the EF_MT_ when all subjects were included in the analysis. A negative correlation means that the thinner the cortex, the stronger the stimulation intensity needed to produce MEPs. Since a thick cortex contains more neurons, a smaller area needs to be activated to stimulate the same amount of neurons, and thus weaker stimulus intensity is sufficient to elicit MEPs.

A similar negative correlation between the cortical thickness and the EF_MT_ corresponding the thenar muscle rMT was found on the precuneus and the cuneus. The precuneus is among other things involved in motor imagery [50], in coordination of motor behaviour [51], [52] as well as in reaction time reduction in a button press task [53]. Furthermore, resting-state functional connectivity analysis has revealed that the precuneus is functionally connected with the motor cortex [54]. In addition, the cuneus is involved in inhibitory control [55]–[57] and also in generating finger movements based on gaze position [52]. Therefore, the precuneus and the cuneus might be involved in some way in cortical excitability and the process of generating MEPs. On the other hand, the primary motor cortex has strong connections to the premotor area which is known to be involved in motor functions. In our study, however, we did not detect any correlation between motor cortex excitability and cortical thickness on the premotor areas. Therefore, since the correlation between the cortical thickness on the precuneus and the cuneus with the sensorimotor cortex excitability was significant in our study population of AD and MCI subjects, it might, at least in part, reflect actual pathophysiological alterations specifically in these areas.

The thicknesses on the cuneus and the precuneus correlated positively with the thickness on the sensorimotor cortex in all groups (Pearson correlation, *p<0.05*). If the correlation with the EF_MT_ was determined solely by the amount of the neurons, the correlations between the cortical thickness and the EF_MT_ would be similar in all ROIs as would be the relationship between the groups as well. In our results, however, this was not the case. The correlation between the EF_MT_ and the cortical thickness varied between the groups especially on the sensorimotor cortex thus implying that there is a difference in the function of the neurons or neuronal circuits in different ROIs between the groups.

Healthy controls displayed no correlation between cortical thickness of the ROIs and EF_MT_. Thus, it seems that normal cortical excitability is not determined solely by the number of excited neurons but instead the healthy brain may have its own individual threshold, depending on local facilitatory and inhibitory interactions. Previous studies have found rMT to be slightly higher in older healthy subjects than in young subjects [38], [58]. One explanation could be that the individual excitability threshold is determined at a young age. Therefore, as a consequence of natural grey matter degeneration related to aging, elderly people have slightly higher motor thresholds than their younger counterparts to counteract the subtle neuronal loss. However, the atrophy rate is slow in normal aging and, thus, there is no direct correlation between the motor threshold and cortical thickness.

In AD subjects the grey matter is clearly atrophied and the severity of the atrophy depends on the progression of the disease. Thus, based on the findings of a negative correlation between EF_MT_ and cortical thickness on the sensorimotor cortex in all of the subjects, one would expect that the EF_MT_ required to elicit a MEP would be highest in the AD group since the cortex is thinnest in AD patients. However, this was not the case in our study, as the mean EF_MT_ of AD patients was lower than that of controls or MCIs although the differences were not statistically significant. Moreover, the correlation between EF_MT_ and cortical thickness in the sensorimotor cortex in the AD group was not statistically significant although it was near the threshold of significance. It has been previously shown that motor cortex excitability is increased in AD patients as compared to controls, i.e. a lower stimulation intensity is required to generate MEPs [9]–[11], [13]. This hyperexcitability has been hypothesized to reflect impairment in both cholinergic activity and abnormal N-methyl-D-aspartic acid transmission [14], [15]. Furthermore, this hyperexcitability has been hypothesized to counterbalance the neuronal loss in the sensorimotor cortex occurring in AD [13], [31]. Our results support the hypothesis of the compensatory mechanism of the hyperexcitability since there was no strong correlation between the EF_MT_ and cortical thickness on the sensorimotor cortex in AD subjects. In MCI subjects, it seems that the motor cortex excitability has not yet increased although the cellular loss has already begun, as indicated by the strong negative correlation between cortical thickness and EF_MT_, especially on the sensorimotor cortex. It has been postulated that cholinergic neural circuits in the motor cortex may be relatively normal in MCI subjects since short afferent inhibition (SAI) is still normal whereas in AD subjects, reduced SAI has been observed [59]. A recent follow-up study examined the cortical excitability after long-term AChEI therapy in AD patients [60]. TMS was established as an objective tool to follow the biological progression of AD. Furthermore, AChEI medication seemed to stabilize both the brain hyperexcitability pattern as well as the cognitive performance of the AD patients. Therefore, the difference in cortical excitability between the MCI and AD patients could be more pronounced if the AD patients were not under AChEI medication. Since the majority of the AD patients in our study were under AChEI medication, the observed differences between the MCI and AD patients cannot thus be explained merely by changes in cholinergic activity.

It has been shown that the motor areas are the last regions to undergo degeneration in AD, whereas cuneus and precuneus cortices are affected at a rather early stage of the disease [61]. In our study, the cortical thickness of both precuneus and cuneus was thinnest in the AD group, although the difference was not statistically significant in our small groups of participants. Furthermore, the cortical thickness of both of these areas had a strong negative correlation with the EF_MT_ in AD subjects. Therefore, it seems that a similar compensatory hyperexcitability for neuronal loss as encountered on the sensorimotor cortex does not occur on the precuneus or the cuneus. In MCI subjects, the thickness of the precuneus correlated with the EF_MT_ implying that some pathophysiological changes might already have occurred on the precuneus. Previous studies have shown that the cortical thickness of the precuneus is lower in multiple-domain MCI subjects, i.e. subjects with both memory impairment and other cognitive deficits, compared to MCI subjects with only memory dysfunction, or when compared to controls [62]. It has been postulated that the atrophy of the precuneus is responsible for the multiple cognitive impairments experienced by MCI subjects. This clear role of the precuneus in neurodegenerative diseases was observed in our results since the negative correlation between the cortical thickness of the precuneus and EF_MT_ was significant both in AD and MCI subjects. A previous study of low-frequency blood oxygenation-level-dependent fluctuations detected decreased coherence in the precuneus in AD patients and this correlated with their MMSE scores [63]. The decrease in the coherence was hypothesized to be related to the resting hypometabolism. In addition, it was found that there is increased coherence in the cuneus in AD subjects suggesting this region might act as a compensatory area for the impaired precuneus. This could at least partly explain why both precuneus and cuneus appeared in our results as two of the areas with a statistically significant negative correlation between cortical thickness and EF_MT_.

### Limitations

One major limitation of our study is that we performed the TMS measurements for the left hemisphere only. The subjects were participants of a TMS study concentrating on EEG responses and the resting motor threshold measurement was therefore an extra recording in the protocol. Since the protocol was rather time consuming, it was necessary to add only a minimum of extra measurements. Therefore, the resting MT was only determined for the right thenar musculature since the majority of subjects were right-handed and thus the correlations were calculated only for the left hemisphere. Another limiting factor might be that we included in the study not only right handed subjects but also a few left-handed and ambidextrous individuals since we did not wish to reduce the group size by including only right-handed subjects. In a previous large-scale normative study there was no significant difference in MT between the dominant and non-dominant hand [38] thus the inclusion of non right-handed subjects and the stimulation of their non-dominant hand should not distort the results of the study. An alternative solution to the handedness issue could have been to perform the measurements for the dominant hand in all subjects, but then we would have faced the problem of combining the thickness results of both hemispheres.

Another limitation in the study is the elapsed time between the MRI scanning and the TMS experiment which for some subjects was several months. We acknowledge that especially in the case of progressive neurodegenerative diseases the timing in the data collection is essential and differences in timing may have an impact on the results. Due to both funding and technical issues we were forced to use older MRI scans when available for the subjects. Fortunately, the majority of the subjects were scanned within a few weeks before the TMS, the average time difference being 2.4 months. Furthermore, the time difference between the measurements was taken into account in the statistical modelling as a covariate of no interest.

### Conclusions

Our results show that the neurodegenerative process proceeds with different dynamics in the structure and function of neuronal circuits from normal conditions via MCI to AD. Our results confirm the hypothesis of the cortical hyperexcitability compensating for the neuronal loss on the sensorimotor cortex in the AD subjects. However, the compensatory mechanism seems to be related only on the sensorimotor cortex since no similar compensation was found on the precuneus or cuneus. Furthermore, this compensatory mechanism does not seem to kick in at early stage of neuronal atrophy since similar mechanism was not found in MCI subjects.
