# Correction: New Insights into Alzheimer's Disease Progression: A Combined TMS and Structural MRI Study

**DOI:** 10.1371/journal.pone.0091271

**Published:** 2014-02-28

**Authors:** The PLOS ONE Staff

Figure 2 is incorrect. The authors have provided a corrected version here.

**Figure 2:**
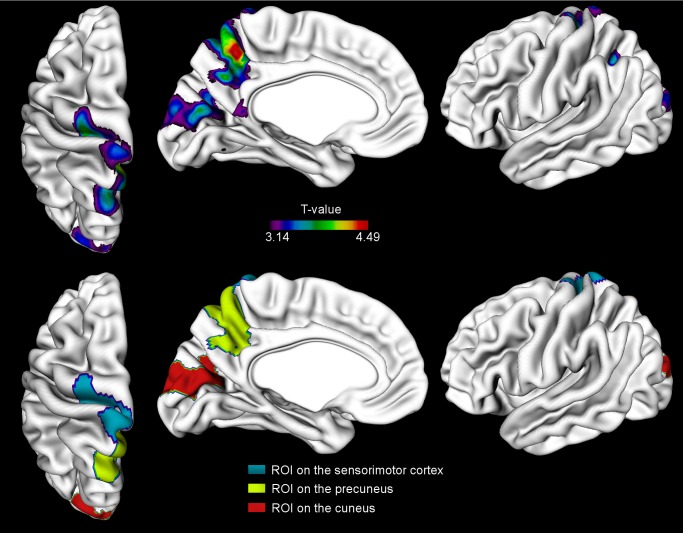
The areas of significant negative correlation between cortical thickness and EFMT for all subjects (p<0.05, FDR-corrected) (upper row) and the corresponding regions of interest with a cluster minimum of 100 nodes (lower row). ROI 1 includes areas on M1 and S1 (in blue), ROI 2 encompasses the precuneus (in yellow) and ROI 3 in the cuneus (in red).
